# Nuclear heat shock protein 110 expression is associated with poor prognosis and chemotherapy resistance in gastric cancer

**DOI:** 10.18632/oncotarget.7821

**Published:** 2016-03-01

**Authors:** Akiharu Kimura, Kyoichi Ogata, Bolag Altan, Takehiko Yokobori, Munenori Ide, Erito Mochiki, Yoshitaka Toyomasu, Norimichi Kogure, Toru Yanoma, Masaki Suzuki, Tuya Bai, Tetsunari Oyama, Hiroyuki Kuwano

**Affiliations:** ^1^ Department of General Surgical Science, Gunma University Graduate School of Medicine, Maebashi, Gunma, Japan; ^2^ Department of Diagnostic Pathology, Gunma University Graduate School of Medicine, Maebashi, Gunma, Japan; ^3^ Department of Digestive Tract and General Surgery, Saitama Medical Center, Saitama Medical University, Kawagoe, Saitama, Japan

**Keywords:** cancer progression, drug resistance, gastric cancer, heat shock protein, heat shock protein 110

## Abstract

Heat shock protein (HSP) expression is induced by the exposure to stress, such as fever, oxidative stress, chemical exposure, and irradiation. In cancer, HSP promotes the survival of malignant cells by inhibiting the induction of apoptosis. In colorectal cancer, a loss-of-function mutation of HSP110 (HSP110ΔE9) has been identified. HSP110ΔE9 inhibits the nuclear translocation of wild-type HSP110, which is important for its chaperone activity and anti-apoptotic effects. The patients carrying HSP110ΔE9 mutation exhibit high sensitivity to anticancer agents, such as oxaliplatin and 5-fluorouracil. There is still insufficient information about HSP110 localization, the clinicopathological significance of HSP110 expression, and its association with chemotherapy resistance in gastric cancer. Here, we found that high nuclear expression of HSP110 in gastric cancer tissues is associated with cancer progression, poor prognosis, and recurrence after adjuvant chemotherapy. *In vitro* results showed that HSP110 suppression increases the sensitivity to 5-fluorouracil and cisplatin of human gastric cancer cell lines. Our results suggest that nuclear HSP110 may be a new drug sensitivity marker for gastric cancer and a potential molecular therapeutic target for the treatment of gastric cancer patients with acquired anticancer drug resistance.

## INTRODUCTION

Gastric cancer is one of the most common cancers worldwide and it is particularly prevalent in Asia [1]. Patients with early-stage gastric cancer have a good prognosis following endoscopic or surgical treatment [2], but advanced or recurrent gastric cancer patients have high mortality rates, due to chemotherapy resistance [3]. Therefore, the investigations of the mechanisms of chemotherapy resistance are necessary, in order to improve patient outcomes.

Heat shock proteins (HSPs) are molecular chaperones that facilitate the proper folding and function of proteins. The expression of HSPs is induced by the exposure to stress, such as fever, oxidative stress, chemical exposure, and irradiation [4, 5]. HSPs provide protection against protein aggregation, facilitate folding of nascent polypeptides, participate in the refolding of proteins that have been damaged, and sequester damaged proteins and target them for degradation [6, 7]. Mammalian HSPs are classified into several protein families based on their molecular weight, namely HSP25/HSP27, HSP40, HSP60, HSP70, HSP90, and HSP110 (also called HSP105) families [8, 9]. HSP70 family proteins are expressed in the cytoplasm and nucleus of mammalian cells [10]. HSP105α and HSP105β, the alternatively spliced products of HSP110 family, are expressed in the cytoplasm (HSP105α) and in nucleus (HSP105β) [11]. Previously, it was reported that nuclear HSPs behave as molecular chaperones in cells [10].

HSPs were shown to be overexpressed in a wide range of human carcinomas, including both solid tumors and hematological malignancies [7, 12, 13]. In cancer, HSP promotes the survival of malignant cells by protecting several oncoproteins from degradation and inhibiting the induction of apoptosis. This suggests that HSP roles are beneficial for cancerous cells and therefore deleterious for cancer patients [14]. High levels of HSPs may correlate with poor prognosis in several types of cancer. For example, high levels of HSP27 were shown to correlate with poor prognosis in ovarian cancer [15], and HSP60 overexpression was correlated with tumor progression and poor prognosis in colon cancer [16] and prostate carcinoma [17]. The elevated expression of HSP70 is associated with poor prognosis in breast [18] and endometrial [19] cancers, while high HSP90 expression is associated with poor prognosis in invasive ductal breast carcinoma [20] but with good prognosis in endometrial cancer [19]. Furthermore, it was reported that various HSPs, including HSP70 and HSP90, are associated with increased chemosensitivity and may represent potential therapeutic targets in refractory malignancies [21–23].

Antitumor response generated by autologous tumor-derived HSP/GRPs (*e.g*., Hsp70, Hsp90, Grp94/gp96, and calreticulin) has been well documented [9, 24, 25]. Studies over the last decade showed that certain tumor-derived HSPs can serve as effective cancer vaccines, and this has been attributed to an HSP-carried peptide antigenic ‘fingerprint’ of the tumor [24, 26–29].

Dorard *et al.* identified a loss-of-function mutation of HSP110 (HSP110ΔE9) in colorectal cancers with microsatellite instability [30]. HSP110ΔE9 lacks a substrate-binding domain, and it is unable to play a role of a molecular chaperone for other HSPs (HSP70 or HSP27). Mutant HSP110ΔE9 protein associates with wild-type HSP110, blocking its translocation into the nucleus and its chaperone functions. Therefore, HSP110ΔE9 overexpression enhances the sensitivity of tumors to anticancer agents, such as oxaliplatin and 5-fluorouracil (5-FU) [30].

However, HSP110 localization, its clinicopathological significance, and its association with chemotherapy resistance in gastric cancer have not been completely elucidated. The objectives of this study were to clarify the significance of HSP110 expression in gastric cancer patients and to assess the effects of HSP110 suppression on chemosensitivity.

## RESULTS

### Clinical significance of nuclear HSP110 expression in gastric cancer patients

**Figure 1:**
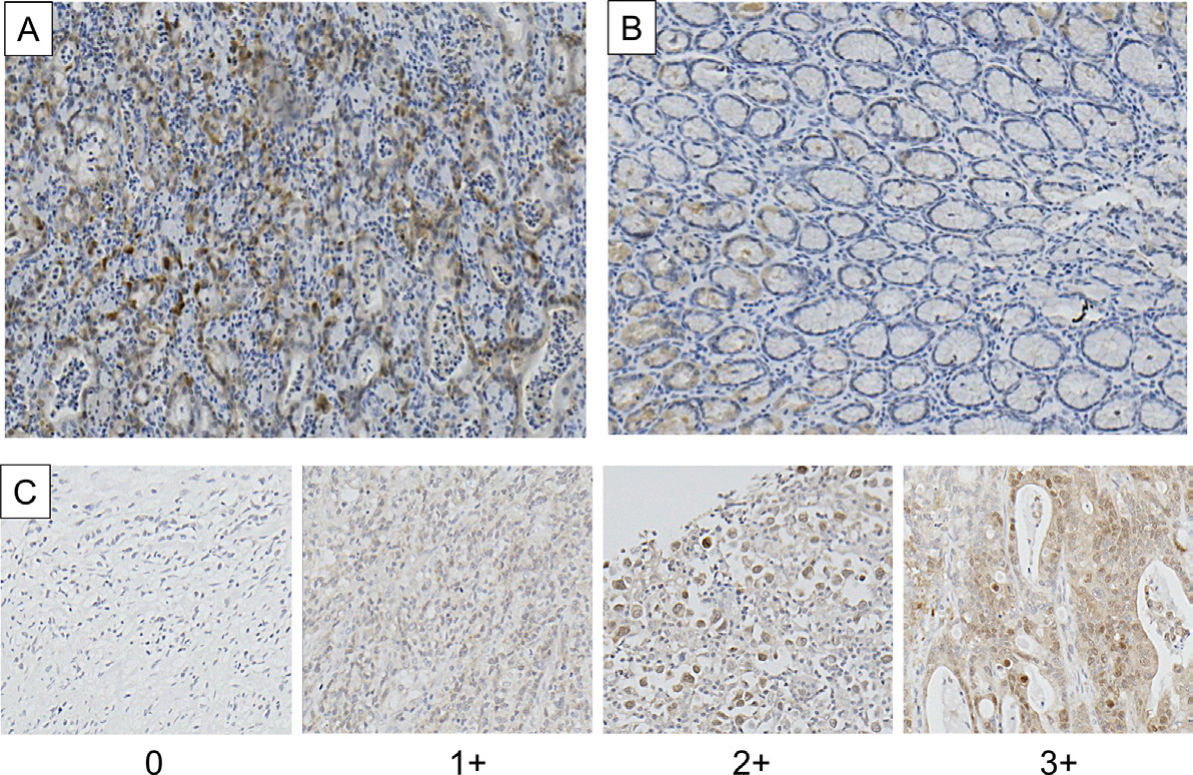
Immunohistochemical staining of HSP110 in primary gastric cancer samples (**A**) Cancerous tissue; (**B**) Non-cancerous tissue (original magnification, × 200). (**C**) Tissue microarray samples (original magnification, 200 ×). The intensity of nuclear HSP110 staining was scored as follows: 0, no staining; 1+, weak staining; 2+, moderate staining; 3+, strong staining.

**Figure 2:**
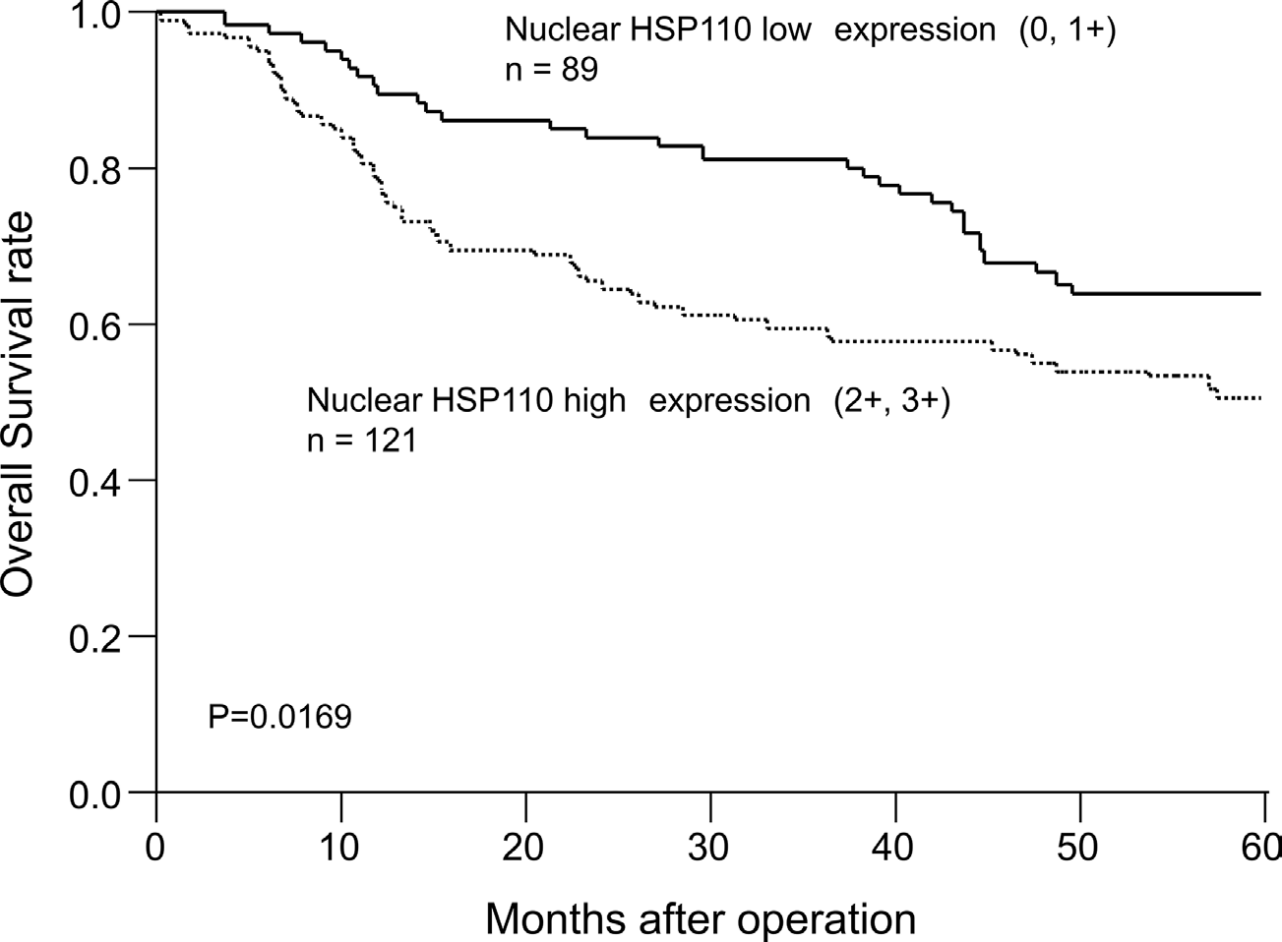
Overall survival of gastric cancer patients according to the nuclear HSP110 expression The overall survival in the nuclear HSP110 high expression group was significantly shorter compared with the low expression group (*P* = 0.0169).

Nuclear HSP110 expression was immunohistochemically evaluated using a tissue microarray that included 210 gastric cancer samples. HSP110 expression in cancer tissues was higher compared with non-cancerous tissues. In the cancer tissues, nucleus and cytoplasm were positive for HSP110 immunostaining (Figure 1A and 1B). In non-cancerous tissues, no or weak staining was observed for HSP110. Nuclear HSP110 expression scores in 210 gastric cancer samples were as follows: 0, 17 (8.1%) samples; 1+, 72 (34.3%) samples; 2+, 80 (38.1%) samples; and 3+, 41(19.5%) samples. Eighty-nine (42.4%) samples were included in the low expression group, and 121 (57.6%) samples were included in the high expression group. The relationship of nuclear HSP110 expression and clinicopathological factors from 210 gastric cancer patients is presented in Table 1. High nuclear expression of HSP110 was significantly associated with venous invasion (*P* = 0.0464). The overall survival rate in the high expression group was significantly lower compared with the low expression group (*P* = 0.0169; Figure 2). Multivariate regression analysis revealed that high expression of nuclear HSP110 is an independent prognostic factor for gastric cancer outcome (*P* = 0.0068), as are the tumor depth (*P* < 0.001) and venous invasion (*P* = 0.0276; Table 2). Additionally, we assessed cytoplasmic HSP110 expression in 210 gastric cancer tissue samples. No significant difference in prognosis was observed between the cytoplasmic HSP110 high expression group and low expression group (*P* = 0.6884, Supplementary Figure S1). Furthermore, additional analysis was performed in order to evaluate the significance of total HSP110 expression in gastric cancer tissues. The high cytoplasmic and nuclear HSP110 expression groups were defined as total HSP110 high expression group (*n* = 74). The low cytoplasmic and nuclear HSP110 expression groups were defined as total HSP110 low expression group (*n* = 73). There was no significant prognostic difference between the total HSP110 high expression group and low expression group (*P* = 0.2021, Supplementary Figure S2). The relationship between total HSP110 expression and clinicopathological factors is shown in Supplementary Table S1. No significant relationships were found between total HSP110 expression and clinicopathological factors, with the only exception being patients' ages.

**Table 1:** The relationship between clinicopathological characteristics of gastric cancer patients and the nuclear HSP110 expression levels

Factors	HSP110 expression in gastric cancer (*n* = 210)		
	Low (*n* = 89)	High (*n* = 121)	*P* value
Age (mean ± standard error)	63.1 ± 1.2	65.7 ± 1.0	0.1007
Gender, *n* (%)			
Male	66 (44.9%)	81 (55.1%)	0.2574
Female	23 (36.5%)	40 (63.5%)	
Histology, *n* (%)			
Well, Moderate	33 (41.8%)	46 (58.2%)	0.8897
Poor, Signet	56 (42.7%)	75 (57.3%)	
Depth, *n* (%)			
sm, mp, ss	48 (40.7%)	70 (59.3%)	0.8266
se, si	41 (44.6%)	51 (55.4%)	
Lymph node metastasis, *n* (%)			
Absent	30 (44.8%)	37 (55.2%)	0.6311
Present	59 (41.3%)	84 (58.7%)	
Lymphatic invasion, *n* (%)			
Absent	9 (47.4%)	10 (52.6%)	0.6459
Present	80 (41.9%)	111 (58.1%)	
Venous invasion, *n* (%)			
Absent	71 (46.7%)	81 (53.3%)	0.0464*
Present	18 (31.6%)	39 (68.4%)	
Stage, *n* (%)			
I	12 (36.4%)	21 (63.6%)	0.5754
II	33 (47.8%)	36 (52.2%)	
III	35 (42.7%)	47 (57.3%)	
IV	9 (34.6%)	17 (65.4%)	

* *P* < 0.05.

Well: well differentiated, Moderate: moderately differentiated, Poor: poorly differentiated, Signet: signet ring cells, sm: submucosa, mp: muscularis propria, ss: subserosa, se: serosa exposed, si: serosa infiltrating.

**Table 2:** Univariate and multivariate analyses of clinicopathological factors affecting overall survival rates after surgery

Clinicopathological variables	Univariate analysis			Multivariate analysis		
	RR	95% CI	*P* value	RR	95% CI	*P* value
Age (*<* 65 years/≥ 65 years)	1.11	0.90–1.37	0.3209	-	-	-
Gender (male/female)	0.9	0.71–1.13	0.3873	-	-	-
Histology (differentiated/undifferentiated)	1.11	0.89–1.38	0.3546	-	-	-
Depth (sm, mp, ss/se, si)	1.83	1.48–2.29	0.0000*	1.74	1.39–2.19	*<* 0.001*
Lymph node metastasis (absent/present)	1.59	1.24–2.11	0.0002*	1.31	0.99–1.78	0.0579
Lymphatic invasion (absent/present)	1.88	1.15–3.79	0.0081*	1.12	0.64–2.33	0.7230
Venous invasion (absent/present)	1.47	1.19–1.83	0.006*	1.29	1.03–1.60	0.0276*
HSP110 expression (low/high)	1.3	1.05–1.63	0.0155*	1.35	1.09–1.70	0.0068*

* *P* < 0.05.

RR: Relative risk, CI: Confidence interval, sm: submucosa, mp: muscularis propria, ss: subserosa, se: serosa exposed, si: serosa infiltrating.

### Prognostic significance of nuclear HSP110 expression in gastric cancer patients who received adjuvant chemotherapy

**Figure 3:**
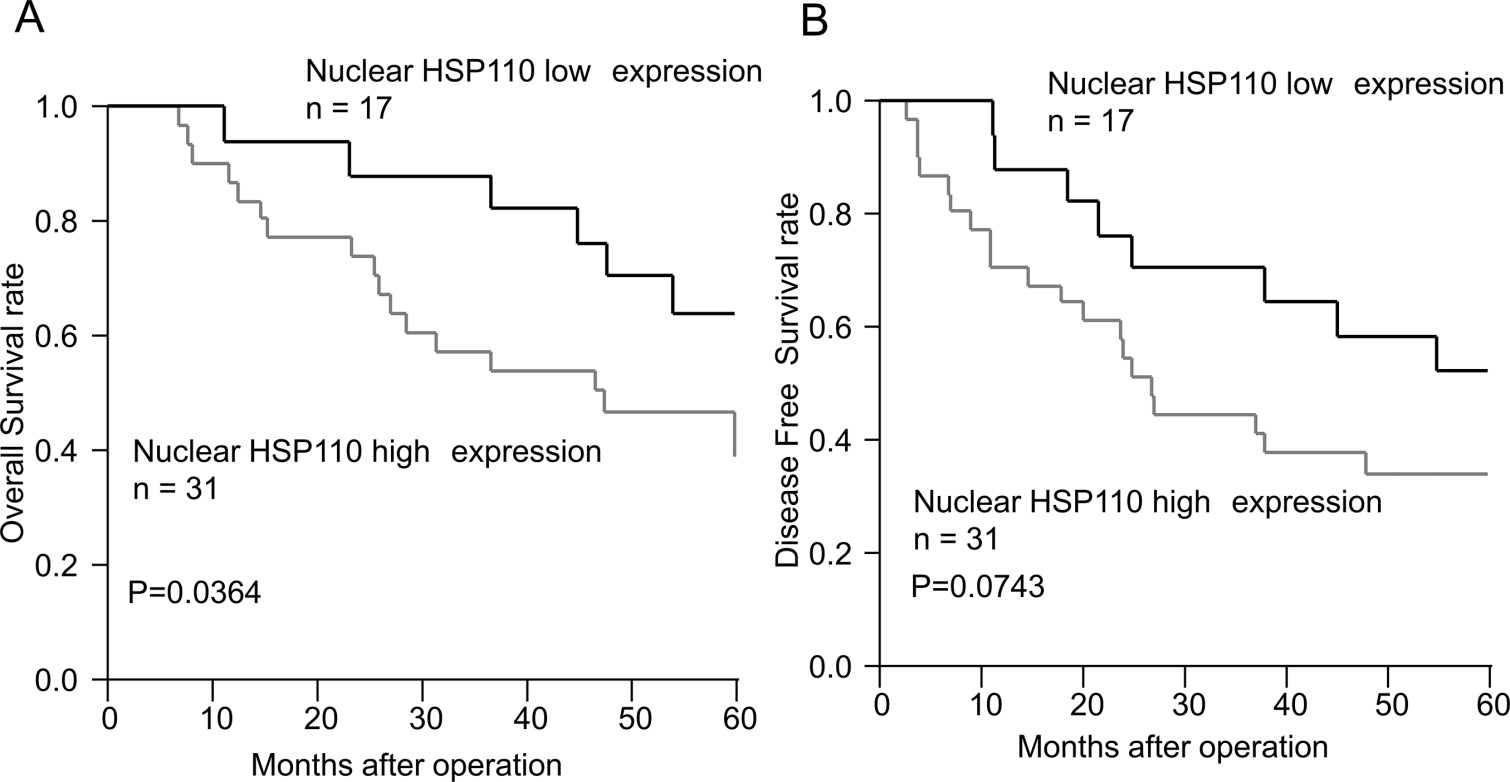
The survival curves of gastric cancer patients who received adjuvant chemotherapy according to nuclear HSP110 expression (**A**) Overall survival. (**B**) Disease-free survival. Among the patients who received adjuvant chemotherapy, the overall survival rate in the nuclear HSP110 high expression group was significantly lower compared with the low expression group (*P* = 0.0364). No significant difference in disease-free survival was observed between these groups; however, the disease-free survival rate in the high expression group tended to be lower compared with the low expression group (*P* = 0.0743).

Forty-eight of the 210 gastric cancer patients received 5-FU-based adjuvant chemotherapy. We evaluated the correlation between nuclear HSP110 expression and prognosis in these patients (Figure 3A and 3B). Among patients who received adjuvant chemotherapy, the overall survival rate in the high expression group was significantly lower compared with the low expression group (*P* = 0.0364). There was no significant difference in disease-free survival between the two groups, but the disease-free survival rate in the high expression group tended to be lower compared with the low expression group (*P* = 0.0743).

### HSP110 expression in gastric cancer cell lines

**Figure 4:**
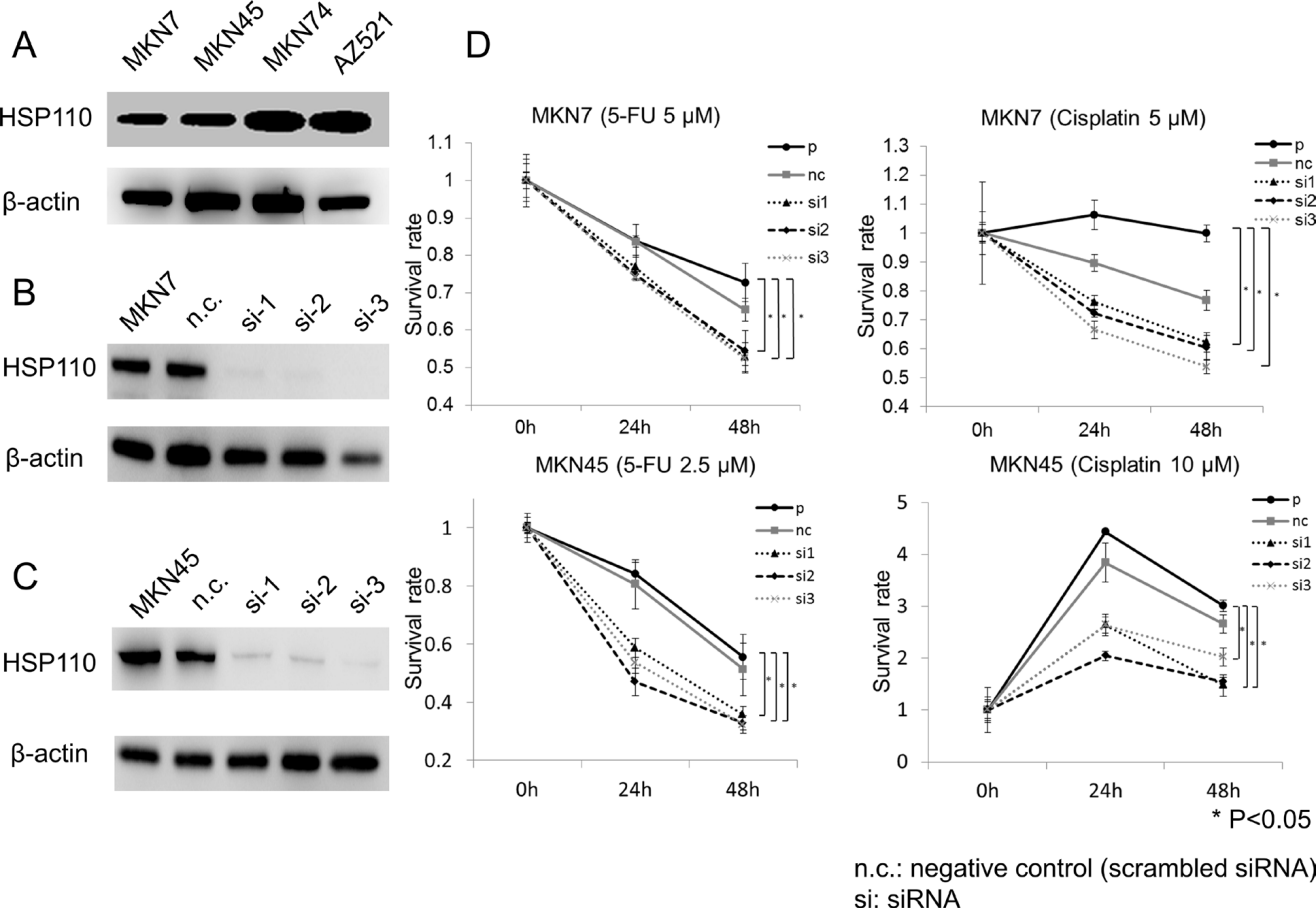
Functional analysis of human gastric cancer cell lines treated with HSP110 siRNA (**A**) The expression of HSP110 in human gastric cancer cell lines was assessed by western blot. β-actin was used as the loading control. (**B**) HSP110 expression was suppressed using HSP110 siRNA (MKN7); (**C**) HSP110 suppression using HSP110 siRNA (MKN45). (**D**) The effects of HSP110 suppression on chemosensitivity of MKN7 and MKN45 cells. Both MKN7 and MKN45 cells showed a significantly increased sensitivity to 5-fluorouracil in HSP110 siRNA-treated groups, compared with the parent and control cells (*p* < 0.05). n.c.: negative control (scrambled siRNA), si: siRNA.

HSP110 expression was detected using western blot in all human gastric cancer cell lines (MKN7, MKN45, MKN74, AZ521) (Figure 4A). MKN7 and MKN45 were further used for the *in vitro* analyses of the effects of HSP110 suppression in gastric cancer cell lines. HSP110 expression was suppressed in MKN7 and MKN45 cells treated with HSP110 siRNA (Figure 4B and 4C).

### The effects of HSP110 suppression on the chemosensitivity of gastric cancer cell lines

We evaluated the correlation between HSP110 suppression and the chemosensitivity of gastric cancer cell lines. Following HSP110 knockdown, MKN7 and MKN45 cells were treated with 5-FU or cisplatin. The sensitivity to 5-FU and cisplatin of HSP110 siRNA-treated cells was significantly higher compared with the parent and control cells (*p* < 0.05; Figure 4D).

## DISCUSSION

Here, we determined that high nuclear expression of HSP110 in gastric cancer tissues is associated with cancer progression and poor prognosis. Among patients who received adjuvant chemotherapy, those included in the high HSP110 expression group showed significantly shorter overall survival compared with the low expression group. *In vitro* study showed that HSP110 suppression increases the sensitivity to 5-FU and cisplatin in human gastric cancer cell lines.

Immunohistochemical analysis demonstrated that the high expression of nuclear HSP110 is associated with poor overall survival. Based on the multivariate analysis of the factors affecting overall survival, the high expression of nuclear HSP110 was shown to be an independent prognostic factor. Previously, it was reported that the high expression of HSP110 is associated with poor prognosis in lung adenocarcinoma [31] and colorectal cancer [32], and our results are consistent with these reports. The chaperoning properties of HSP110 are integral to the ability of these molecules to modulate immune functions and for the development of large chaperone complex vaccines for cancer immunotherapy [9]. Nakajima *et al.* reported that high cytoplasmic HSP110 expression induces CD4+ T lymphocyte infiltration, which was shown to be associated with good prognosis in esophageal cancer [33]. We assessed total and cytoplasmic HSP110 expression in gastric cancer samples, but no significant prognostic differences were observed in the total and cytoplasmic HSP110 expression between the high and low groups (Supplementary Figures S1, S2). It was previously reported that wild-type nuclear, rather than cytoplasmic, HSP110 prevents the induction of apoptosis in colorectal cancer cells [30]. Therefore, we suggest that nuclear HSP110 expression levels may be a useful prognostic and drug sensitivity marker for gastric cancer.

In this study, the high expression of nuclear HSP110 was shown to be associated with venous invasion (Table 1). HSPs promote cancer progression in several cancer types, and Gong *et al.* [34] reported that the invasion potential of hepatocarcinoma cells is increased by HMGB1-induced tumor NF-κB signaling, through the activation of HSP70. Sims *et al.* [35] reported that extracellular HSP70 and HSP90α contribute to the matrix metalloproteinase-2 activation and breast cancer cell migration and invasion. It has also been reported that HSP110 is co-expressed with HSP70 and HSP90 during stress, and that it promotes HSP90 activity and may function as a nucleotide exchange factor for cytosolic HSP70 [36]. We elucidated whether HSP110 expression can facilitate cancer invasion through the activation of HSP70 and HSP90.

Hosaka *et al.* [37] reported that HSP110 suppression induces apoptosis in cancer cell lines but not in fibroblasts. Dorard *et al.* [30] identified a loss-of-function mutation of HSP110 (HSP110ΔE9) in colorectal cancer with microsatellite instability. HSP110ΔE9 overexpression enhanced cancer cell sensitivity to anticancer agents. Here, low nuclear HSP110 expression group had better prognosis compared with the high expression group, and HSP110 suppression was shown to increase cell sensitivity to 5-FU and cisplatin in human gastric cancer cell lines, which is consistent with the previous reports. Novel treatment strategies, combining an HSP110 inhibitor and an anticancer agent, may be effective for the treatment of gastric cancer patients with acquired anticancer drug resistance.

Previously, HSP110 was identified as a cancer antigen in various human carcinomas [31, 32]. We report here that the high expression of the nuclear HSP110 was observed in gastric cancer patients. Wang *et al.* [29] developed a vaccine composed of a recombinant protein coupled with large heat shock protein. Our results suggest that chemosensitivity may decrease due to heat stress-induced HSP110 expression. Therefore, various vaccines, which may utilize HSPs (*i.e.,* covalent coupling, isolation of HSPs with antigens attached, recombinant vaccines made by heat-denaturation of full-length antigens and HSP110) may be useful in anticancer treatments.

HSP110-specific siRNAs were used to suppress the expression of HSP110 in gastric cancer cell lines, which presents a limitation of this study. Therefore, total HSP110 expression was suppressed, and not only the specific nuclear HSP110 expression. Total HP110 expression was strongly suppressed in MKN7 and MKN45 by the HSP110-specific siRNA, but this is probably the consequence of the suppression of both nuclear and cytoplasmic HSP110 expression.

In conclusion, the high expression of nuclear HSP110 was shown to be associated with cancer progression, poor prognosis, and recurrence after adjuvant chemotherapy in gastric cancer patients. Furthermore, HSP110 suppression increased the sensitivity to 5-FU and cisplatin in the human gastric cancer cell lines. Our results suggest that nuclear HSP110 expression in gastric cancer may be a new prognostic and drug sensitivity marker, and HSP110 may serve as a new molecular therapeutic target for the treatment of refractory gastric cancer.

## METHODS

### Patients and samples

Primary gastric cancer tissues were obtained from gastric cancer patients (*n* = 210; 147 men and 63 women) who underwent radical gastrectomy at the Department of General Surgical Science, Gunma University Hospital, Japan, between January 1999 and May 2006. The stage of gastric cancer was described according to the classification of gastric carcinoma of the Japanese Gastric Cancer Association's 3rd English edition [38]. Forty-eight patients received 5-FU-based adjuvant chemotherapy between January 2003 and May 2006. The correlation between HSP110 expression and clinicopathological factors and prognosis was evaluated in these patients. Written informed consents were obtained from all patients according to institutional guidelines.

### Tissue microarray analysis and immunohistochemical staining

Tumor samples were fixed in formalin, embedded in paraffin, and stored in the archives of the Clinical Department of Pathology, Gunma University Hospital, Japan. For 210 gastric cancer patients, one paraffin block containing representative non-necrotic tumor areas was selected, and gastric cancer tissue cores (2.0 mm diameter per tumor) were sampled from the representative areas and transferred into the paraffin block using a tissue arraying instrument (Beecher Instruments, Silver Spring, MD, USA). Cores were arranged into quad tissue array blocks, with each containing 50–55 tumor cores. Tissue microarray blocks were cut into 3.5-μm thick sections, and were used for the subsequent immunohistochemical staining. Additionally, 4-μm sections were cut from the paraffin blocks of 10 gastric cancer samples, selected among 210 gastric cancer patients for validation.

All sections were incubated at 60°C for 60 min and deparaffinized in xylene. Afterward, these sections were rehydrated and incubated with fresh 0.3% hydrogen peroxide in 100% methanol for 30 min at room temperature, in order to block endogenous peroxidase activity. Following the rehydration through a graded series of ethanol treatments, the sections were heated in boiling water and soaked in Immunosaver (Nishin EM, Tokyo, Japan) at 98°C for 90 min. Non-specific binding sites were blocked by incubating the sections with Protein Block Serum-Free (DAKO, Carpinteria, CA, USA) for 30 min. A rabbit monoclonal anti-HSP110 antibody (GeneTex, CA, USA) was applied at 1:100 dilution, for 24 h at 4°C. The primary antibody was visualized using the Histofine Simple Stain MAX-PO (MULTI) (Nichirei, Tokyo, Japan) according to the instruction manual. A chromogen, 3,3-diaminobenzidine tetrahydrochloride, was applied as a 0.02% solution containing 0.005% hydrogen peroxide in 50 mM ammonium acetate-citrate acid buffer (pH 6.0). The sections were lightly counterstained with Mayer's hematoxylin and mounted. The evaluation of immunohistochemical staining was performed by two independent researchers who were blinded to the patients' data. We focused on nuclear HSP110 expression, and the intensity of nuclear HSP110 staining was scored as follows: 0, no staining; 1+, weak staining; 2+, moderate staining; 3+, strong staining. Gastric cancer patients were assigned to the nuclear HSP110 low expression group (0, 1+) or high expression group (2+, 3+), according to staining score (Figure 1C). Additionally, the tissues adjacent to the cancerous tissues in the tissue microarray samples were considered non-cancerous tissue. We evaluated the expression of HSP110 in the non-cancerous tissue of these 10 samples, for validation. The non-cancerous tissue was defined as the normal gastric mucosa tissue or stromal cells.

### Cell culture

The human gastric cancer cell lines MKN7, MKN45, MKN74, and AZ521 were used in this study. These cell lines were obtained from RIKEN BRC through the National Bio-Resource Project of MEXT, Tokyo, Japan. The cells were cultured in RPMI 1640 medium (Wako, Osaka, Japan) supplemented with 10% FBS and 1% penicillin-streptomycin (Invitrogen, Carlsbad, CA, USA).

### siRNA transfection

HSP110-specific siRNA was purchased from Bonac Corporation (Fukuoka, Japan). MKN7 and MKN45 cells were plated at a density of 1.0 × 10^6^ cells per well in 100 μl of Opti-MEM I Reduced Serum Medium (Invitrogen, Carlsbad, CA, USA). Twenty nM of HSP110-specific siRNA 1, 2, 3 and scrambled siRNA (negative control) were added to the cells, and cells were transfected with siRNAs using an electroporator (CUY-21 EDIT II; BEX, Tokyo, Japan), according to the manufacturer's instructions. Poring pulses were applied at 125 V (pulse length, 10.0 ms; 1 pulse; interval, 40.0 ms), and transfer pulses were applied at 10 V (pulse length, 50.0 ms; 5 pulses; interval, 50.0 ms). After 72 h of incubation, further experiments were performed.

### Protein extraction and western blot analysis

Western blotting was performed to confirm the expression of HSP110 and β-actin in gastric cancer cell lines. Transfected cells were incubated for 72 h, and total proteins were extracted from MKN7, MKN45, MKN74, and AZ521 cells using PRO-PREP Protein Extraction Solution Kit (iNtRON Biotechnology, Sungnam, Kyungki-Do, Korea). The proteins were separated on 4–12% Bis-Tris Mini Gels (Life Technologies Corporation, Carlsbad, CA, USA), and transferred to membranes using an iBlot Dry Blotting System (Life Technologies Corporation, Carlsbad, CA, USA). The membranes were incubated overnight at 4°C with rabbit monoclonal anti-HSP110 antibody (1:1000; GeneTex, CA, USA) and anti-β-actin antibody (1:1000; Sigma-Aldrich, St Louis, MO, USA). Following this, the membranes were incubated with horseradish peroxidase-conjugated anti-rabbit secondary antibodies, and the target proteins were detected with the ECL Prime Western Blotting Detection System (GE Healthcare, Tokyo, Japan) using Image Quant LAS4000 (GE Healthcare Life Sciences, UK).

### Chemosensitivity assay

Water-soluble tetrazolium-8 (Cell Counting Kit-8; Dojindo Laboratories, Japan) was used in order to evaluate the sensitivity to cisplatin and 5-FU. After 72 h of incubation following the transfection, MKN7 and MKN45 cells were seeded (1 × 10^4^ cells/well) into 96-well plates in 100 μl of RPMI 1640 medium containing 20% FBS before drug exposure. After 24 h of pre-incubation, 10 μl of Cell Counting Kit-8 reagent were added, and the cells were additionally incubated for 2 h at 37°C. The absorbance of each well was detected at 450 nm using an xMark Microplate Absorbance Spectrophotometer (Bio Rad, Hercules, CA, USA). Afterward, the cells were treated with various concentrations of cisplatin and 5-FU for 48 h. Viability was determined using colorimetry by measuring absorbance every 24 h.

### Statistical analysis

Data for continuous variables were expressed as mean ± standard error of the mean. Significance was determined using Student's *t*-test and analysis of variance. The statistical analysis of the immunohistochemical staining results was performed using the chi-squared test. Survival curves were generated according to the Kaplan-Meier method and analyzed using the log-rank test. Prognostic factors were examined by univariate and multivariate analyses using a Cox proportional hazards model. Results were considered statistically significant when *P* value was *<* 0.05. All statistical analyses were performed using JMP software, version 12 (SAS Institute Inc., Cary, NC, USA).

## SUPPLEMENTARY MATERIALS TABLE AND FIGURES


