# Supplementary material for: Nuclear heat shock protein 110 expression is associated with poor prognosis and chemotherapy resistance in gastric cancer
Source: Oncotarget. 2016 Mar 1;7(14):18415–23. doi: 10.18632/oncotarget.7821 (PMC4951298; doi:10.18632/oncotarget.7821)
Supplement: Supplementary file 1 [file oncotarget-07-18415-s001.pdf]

Nuclear heat shock protein 110 expression is associated with poor prognosis and chemotherapy resistance in gastric cancer

Supplementary Materials

Supplementary Table S1: Clinicopathological characteristics of gastric cancer patients according to total HSP110 expression

Factors	total HSP110 expression in gastric cancer (n = 147)		
	Low (n = 73)	High (n = 74)	P value
Age (mean ± standard error)	62.8 ± 1.3	66.0 ± 1.3	0.0433*
Gender, n (%)			
Male	52 (51.5%)	49 (48.5%)	0.5117
Female	21 (45.7%)	25 (54.3%)	
Histology, n (%)			
Well, Moderate	28 (47.5%)	31 (52.5%)	0.6619
Poor, Signet	45 (51.1%)	43 (48.9%)	
Depth, n (%)			
sm, mp, ss	40 (44.9%)	49 (55.1%)	0.1561
se, si	33 (56.9%)	25 (43.1%)	
Lymph node metastasis, n (%)			
Absent	25 (51.0%)	24 (49.0%)	0.8155
Present	48 (49.0%)	50 (51.0%)	
Lymphatic invasion, n (%)			
Absent	8 (47.1%)	9 (52.9%)	0.8195
Present	65 (50.0%)	65 (50.0%)	
Venous invasion, n (%)			
Absent	59 (54.1%)	50 (45.9%)	0.0856
Present	14 (37.8%)	23 (62.2%)	
Stage, n (%)			
I	10 (38.5%)	16 (61.5%)	0.6156
II	26 (53.1%)	23 (46.9%)	
III	29 (52.7%)	26 (47.3%)	
IV	8 (47.1%)	9 (52.9%)	

*$P < 0.05$.
Well: well differentiated, Moderate: moderately differentiated, Poor: poorly differentiated, Signet: signet ring cell, sm: submucosa, mp: muscularis propria, ss: subserosa, se: serosa exposed, si: serosa infiltrating.

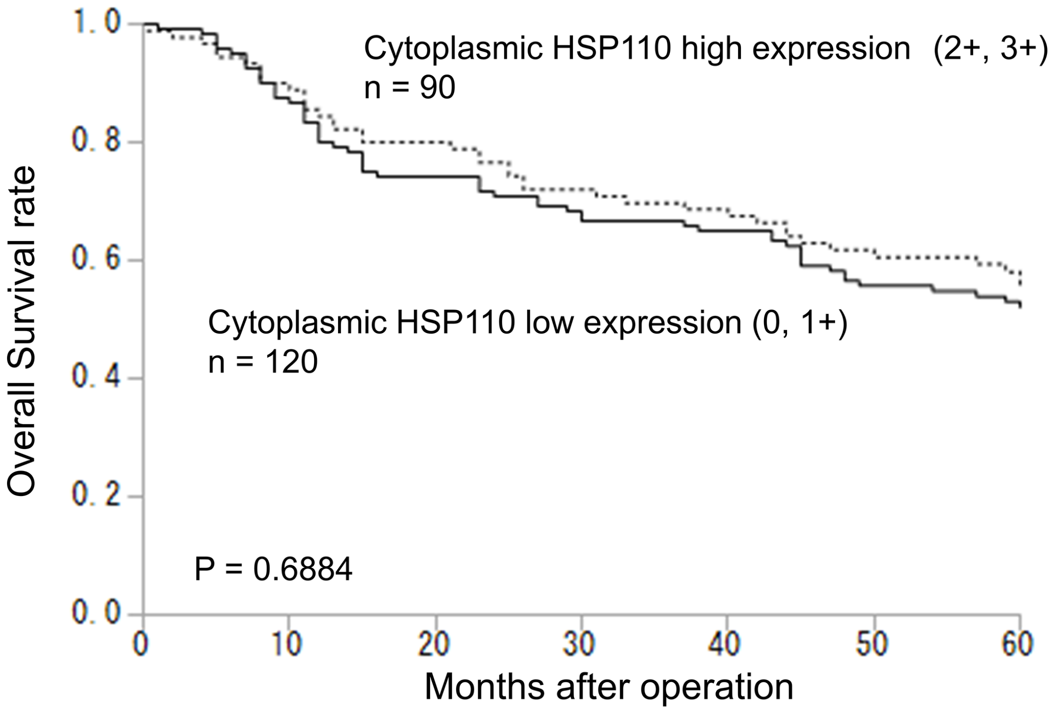

Supplementary Figure S1: Overall survival of gastric cancer patients according to the cytoplasmic HSP110 expression. The intensity of cytoplasmic HSP110 staining was scored as follows: 0, no staining; 1+, weak staining; 2+, moderate staining; 3+, strong staining. We divided 210 gastric cancer patients into the cytoplasmic HSP110 low expression group (0, 1+) and high expression group (2+, 3+), according to this score. No significant difference in prognosis was observed between these groups (*P* = 0.6884).

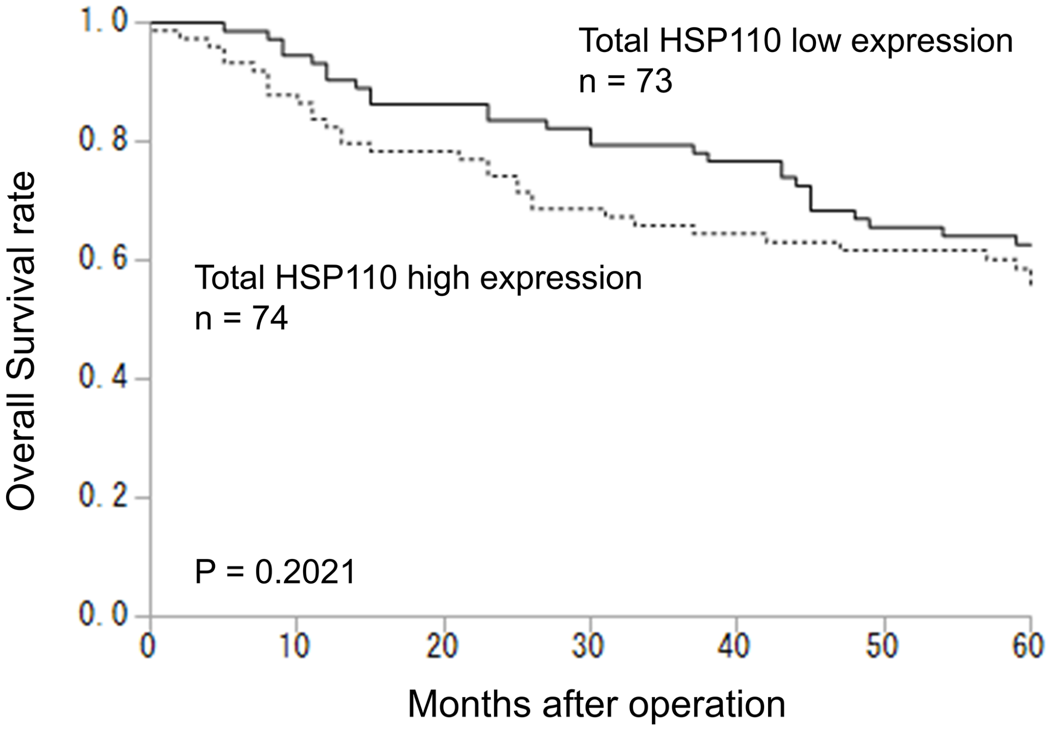

Supplementary Figure S2: Overall survival of gastric cancer patients according to total HSP110 expression. High cytoplasmic and nuclear HSP110 expression groups were defined as total HSP110 high expression group (*n* = 74). Low cytoplasmic and nuclear HSP110 expression groups were defined as total HSP110 low expression group (*n* = 73). No significant prognostic difference was observed between the total HSP110 high expression group and low expression group (*P* = 0.2021).
